# Modelling the potential for parenting skills interventions to reduce inequalities and population prevalence of children's mental health problems: Evidence from the Millennium Cohort Study

**DOI:** 10.1016/j.ssmph.2021.100817

**Published:** 2021-05-13

**Authors:** Steven Hope, Anna Pearce, Mario Cortina-Borja, Catherine Chittleborough, Jane Barlow, Catherine Law

**Affiliations:** a UCL Great Ormond Street Institute of Child Health, London, United Kingdom; b MRC/CSO Social and Public Health Sciences Unit, University of Glasgow, Glasgow, United Kingdom; c School of Public Health, University of Adelaide, Adelaide, Australia; d Department of Social Policy and Intervention, University of Oxford, Oxford, United Kingdom

**Keywords:** Parenting skills, Child mental health problems, Simulated interventions, Inequalities, Cohort research

## Abstract

Parenting programmes can improve parenting quality and, in turn, children's mental health. If scaled-up, they have the potential to reduce population inequalities and prevalence in child mental health problems (MHP). However, this cannot be investigated with trials. Using data from the UK Millennium Cohort Study (18,000 children born 2000–2002), we simulated population impact of scale-up of seven parenting programmes. Predicted probabilities of child MHP (Strengths and Difficulties Questionnaire) by household income quintile (Risk ratios [RRs] and differences [RDs], 95% confidence intervals [CI]) were estimated from logistic marginal structural models, adjusting for parenting quality scores (Child-Parent Relationship Scale at 3 years) and confounders. The impact of scaling-up parenting programmes was simulated by re-estimating predicted probabilities of child MHP after increasing parenting scores according to intervention intensity, targeting mechanisms and programme uptake levels. Analyses included data from 14,399 children, with survey weights and multiple imputation addressing sampling design, attrition and item missingness. Prevalence of child MHP at 5 years was 11.3% (11.4% unadjusted), with relative and absolute income inequalities (RR = 4.8[95%CI:3.6–5.9]; RD = 15.8%[13.4–18.2]). In simulations, universal, non-intensive parenting programmes reduced prevalence (9.4%) and absolute inequalities (RR = 5.0[95%CI:3.8–6.2]; RD = 13.6%[11.5–15.7]). Intensive programmes, targeting a range of potential risk criteria (e.g. receipt of means-tested benefits), reduced inequalities (RR = 4.0[95%CI:3.0–4.9]; RD = 12.4%[10.3–14.6] and, to a lesser extent, prevalence (10.3%). By simulating implementation of parenting programmes, we show that universal non-intensive and targeted intensive approaches have the potential to reduce child MHP at population level, and to reduce but not eliminate inequalities, with important implications for future policy and practice.

## Highlights

• We simulated the potential impact on child MHP prevalence and inequalities of scaling up parenting skills interventions.
• Results suggest that universal parenting programmes might reduce child MHP prevalence.
• Additional targeted, intensive interventions would be necessary to reduce inequalities.

## 1 Introduction

Mental health problems (MHP) such as emotional and behavioural difficulties, are common among children in the UK (Green et al., 2005). They can begin early, with the prevalence of mental disorder in UK preschool age children in 2017 estimated to be 6% (Vizard et al., 2018). As with MHPs in older children, there are also high levels of continuity, with studies showing between 50% and 80% stability for preschool age children over periods ranging from 1 to 6 years (Bilancia & Rescorla, 2010; Briggs-Gowan et al., 2006). The negative impact of exposure to economic hardship on child MHP is well-established. A review of 55 studies, found that 52 studies showed greater risks of MHP among socioeconomically disadvantaged children and adolescents compared to their economically advantaged peers (Reiss, 2013). Such inequalities in MHP are already apparent in preschool years and persist throughout childhood (Rougeaux et al., 2017), and the removal of inequalities would mean that children from socioeconomically disadvantaged groups were no more likely than those from more advantaged groups to experience MHPs.

Parenting has been identified as being one of the key factors influencing child MHP, and in particular, the development of externalising (i.e. behavioural) problems. A recent review based on 1435 studies, found for example, that harsh control, psychological control, authoritarian, permissive, and neglectful parenting were associated with an increase in externalising problems (Pinquart, 2017). While the mechanisms by which socioeconomic circumstances influence child mental health are diverse and complex (Straatmann et al., 2019), adverse parenting quality is one pathway that links disadvantage with child MHP. A number of studies have shown that the stresses on parents resulting from the experience of economic hardship are associated with adverse parenting practices and subsequent child MHP (Conger et al., 2010; Linver et al., 2002; Mazza et al., 2016; Schoon et al., 2010). Interventions that focus on developing positive parenting practices therefore have the potential to reduce both prevalence and inequalities in child MHP, and a number of parenting programmes, mostly based on social learning theory and behavioural principles in which parents are taught to reward positive behaviours and ignore negative behaviours, have been developed for use both on a universal (e.g. Level 1 Triple P) and targeted (e.g. Levels 2–5 Triple P; Early Years Program) basis. The primary aim of such programmes is to support parents to provide the type of parenting practices that are recognised to be associated with optimal social, emotional and behavioural development in children. These programmes range in intensity from web or group-based guidance to extended one-to-one training, and are delivered in various clinical, community-based or virtual settings, by a range of practitioners (e.g. health visitors; psychologists; trained volunteers).

Standardised parenting programmes have been demonstrated to be effective in improving mental health outcomes for young (e.g. 0–3 years) (Barlow et al., 2016) and older (e.g. 3–12 years) (Furlong et al., 2012) children, in addition to improving aspects of parental functioning (for example, anger, stress, anxiety, guilt, confidence, and satisfaction with the partner relation) in the short-term (Barlow et al., 2014). A number of attachment-based interventions (e.g. using video feedback) have also been found to be effective in improving parental sensitivity and attachment during the very formative years of childhood (Bakermans-Kranenburg et al., 2003). A meta-analysis of individual participant data from a number of RCTs has shown that parenting programmes are associated with improved parenting practices and reduced child disruptive behaviour, with consistent improvements in child behaviour across social groups (Gardner et al., 2017).

Thus, the findings from trials suggest that parenting programmes, if implemented at scale in the population, might be effective at improving parenting and child MHP. Nevertheless, there are criticisms of aspects of the trial evidence, in terms of sample size, study design, and the quality of data collected (Coyne & Kwakkenbos, 2013; Wilson et al., 2012). Trial samples are generally neither large nor representative enough to accurately estimate either sub-group or population effectiveness. Heterogeneity in trial design and evaluation may influence the level of effectiveness demonstrated. Trials are also limited in their ability to show the impact of parenting programmes on inequalities in child MHP, as they focus on effectiveness rather than access to an intervention. Trials seldom investigate how to identify families who would benefit from parenting skills interventions, and there is little evidence about what successfully works in terms of targeting services in the early years (National Institute of Health and Care Excellence, 2012). The limited evidence from large-scale evaluations of services designed to support families and children in the UK is mixed. While the evaluation of Sure Start programmes showed improved parenting and developmental outcomes in the early years (Belsky et al., 2006; Melhuish et al., 2008), the evaluation of the Troubled Families Programme (Day et al., 2016) did not provide clear evidence of its effectiveness. Ultimately, such evaluations show the results of policy decisions already taken.

Government advisers have suggested that the availability of parenting skills training as part of service provision would likely benefit child mental health at a population level (Davies, 2013; National Institute of Health and Care Excellence, 2012). However, the existing evidence base provides limited insights about what would happen should policies on provision of parenting programmes be scaled up and provided at population level in the “real world”, as part of routine services. Simulations of interventions (“What if” scenarios) provide an opportunity to model different policy options in terms of the impact of targeting, intensity and uptake of parenting programmes on population prevalence and inequalities in child MHP before implementation.

Child MHP is prevalent and socially patterned, and parenting practices have been identified as a potential factor to tackle this, through a national roll out of parenting skills programmes. In the absence of population-level evidence, we aimed to model the impacts of hypothetical parenting skills intervention scenarios using a simulation approach carried out within a mediation framework (Chittleborough et al., 2014) applied to nationally-representative data on children born in the UK at the start of the new millennium. Specifically, we estimated whether changes to parenting skills in parents of pre-school age children (the mediator between socio-economic circumstances and child MHP, manipulated to reflect the potential impact of hypothetical interventions) would reduce the prevalence of child MHP at age 5, and narrow the gap in prevalence between less and more advantaged groups (that is, reduce inequalities). Scenarios were informed by parenting intervention evidence and reflected different potential policy options, including level of programme intensity (or effectiveness), targeting of eligibility for the intervention, and level of uptake of the intervention. Thus, the scenarios modelled ranged from universal provision of a low-intensity pre-school parenting skills intervention to intensive interventions targeted toward particular groups of individuals.

## 2 Methods

### 2.1 Subjects and design

We used data from the Millennium Cohort Study (MCS), a longitudinal study of children born in the UK between September 2000 and January 2002, which has been described elsewhere (Connelly & Platt, 2014). The first study contact with the cohort child was at around age 9 months, with survey interviews carried out by trained interviewers in the home with the main respondent (usually the mother) and their partner, where present. Information was collected on 18,818 infants (of which our analyses were restricted to 18,296 singletons). We used data from the initial survey and those carried out subsequently at ages three (n = 15,381) and five (n = 15,041). Data were obtained from the UK Data Archive, University of Essex in March 2014. Ethical approval for the MCS was received from a Research Ethics Committee at each sweep (Hansen, 2014).

### 2.2 Measures

#### 2.2.1 Child mental health problems (SDQ)

At 5 years, mental health problems were assessed using the Strengths and Difficulties Questionnaire (SDQ) (Goodman, 1997), a 25-item measure completed by the parent. We used the total difficulties score, the sum of four difficulties scales (peer problems, conduct disorders, hyperactivity and emotional problems) to classify children, using validated cut-offs, for ‘normal’ (0-13), or ‘borderline-abnormal’ scores (14–40). Sensitivity analyses involved repeating analyses using separate subscale scores for Internalising (emotional problems and peer problems) and Externalising (hyperactivity and conduct disorders) behaviour. Results for these sensitivity analysis (not shown) were similar to those reported here for the SDQ total difficulties score.

#### 2.2.2 Socio-economic circumstances (SECs)

Socio-economic inequalities in SDQ were measured according to quintile of equivalised household income, reported at 9 months. We repeated the analyses using an alternative measure of SECs (maternal highest educational qualification, dichotomised as ‘low’ [<GCSEs A*-C] versus ‘high’ [GCSEs A*-C] and the pattern of results was similar to that for income [Appendix A: Table A1]).

#### 2.2.3 Parenting quality

Parenting quality was measured using the Pianta Child-Parent Relationship Scale (CPRS: Short-Form), completed by the parent when the child was aged 3 years (Johnson et al., 2015). The scale comprises 15 items assessing the parent's feelings and beliefs about their relationship with the child, and the child's behaviour to the parent. The two dimensions of the scale Conflicts (8 items; reverse-scored) and Closeness (7 items) were summed to produce a total score, reflecting the extent to which there is a positive relationship between parent and child (scoring range 30–75). As sensitivity analyses, we repeated analyses using separate scores for Conflicts and Closeness subscales. Patterns of results were similar to those reported here for the overall score (not shown).

#### 2.2.4 Confounders

**Fig. 1:**
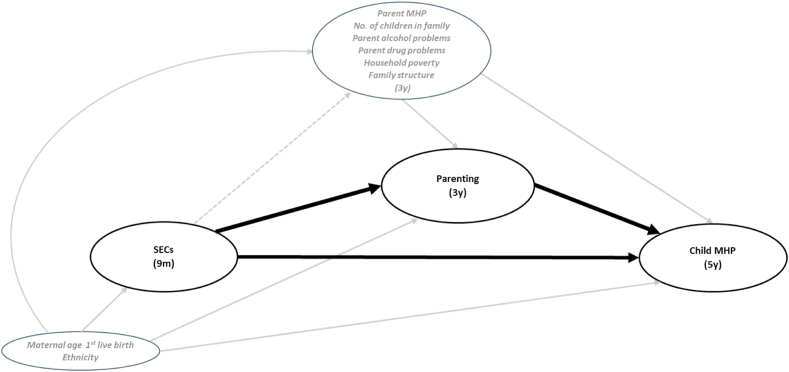
Directed Acyclic Graph (DAG) of the hypothesised association between socio-economic circumstances (SECs), parenting quality and child mental health problems (MHP) Dotted line shows that while there is a causal relationship between the exposure and time-varying confounders, this pathway can be left open when using marginal structural models, so as not to underestimate the direct effect of SECs on Child MHP acting via these factors (which were not mediators of interest).

We accounted for the following factors, which were identified as potential confounders, as guided by a Directed Acyclic Graph of the hypothesised association between socio-economic circumstances, parenting quality and child mental health problems (Fig. 1).

Two potential baseline confounders were included: ethnicity (White, Mixed, Indian, Pakistani and Bangladeshi, Black or Black British, and Other) and maternal age at first live birth (14–19, 20–29, 30–39 and 40 or more years), both recorded at 9 months, as they may influence both socio-economic circumstances and child behaviour. Six potential intermediate confounders of the relationship between parenting quality and child behaviour were accounted for (reported at 3 years): family structure (two natural parents, reconstituted, or lone parent family), the number of children in the household (1 child, 2–3 children, and 4 or more children), parental alcohol problems (one or both parents reporting that they drank every day or had a drink in morning to steady their nerves), parental drug use (one or both parents reporting that they used recreational drugs regularly), parental mental health problems (one or both parents obtaining high score [13 or more] on the Kessler-6 scale (Kessler et al., 2003)) and household income poverty (equivalised family income below 60% of the national median).

#### 2.2.5 Sex

There was no evidence that income inequalities in child MHP at 5 years varied by sex (*p = 0.5*), that income inequalities in parenting quality varied by sex (*p = 0.2*), or that the impact of parenting on child MHP varied by sex (*p = 0.4*). Therefore, results are presented for boys and girls combined.

### 2.3 “What if?” intervention scenarios

A series of “What if?” intervention scenarios were modelled, reflecting potential intervention and targeting strategies, summarised in Table 1. The observed parenting quality variable was manipulated to simulate increases in parenting scores that might be achieved through parenting skills interventions, modelled as though offered to all families or targeted to specific groups. There is a lack of evidence on population parenting programmes. The degree to which the parenting quality variable was manipulated was informed by evidence from trials and systematic reviews of the trial evidence. The evidence from evaluations of parenting programmes is heterogeneous, and we chose effect sizes that balanced the evidence-base and a hypothetical position, asking what might happen to prevalence and inequalities in child MHP if parenting interventions were rolled out across the population, either intensively or non-intensively. We modelled two effect sizes, both within the ranges identified in the literature (Barlow et al., 2014; Furlong et al., 2012; Lindsay et al., 2010; Nowak & Heinrichs, 2008): a 0.4SD increase in parenting scores that might follow a universal, non-intensive intervention; or a 0.9SD increase in parenting scores after an intensive intervention only offered to small subsets of the population. In recognition that an effect size of 0.9SD is large, a sensitivity analysis also modelled an effect size of 0.6SD in scenarios with intensive interventions.

**Table 1:** Summary of modelled intervention scenarios.

Intervention scenario	Average effectiveness	Eligibility	Uptake
1. Universal	+0.4SD parenting skills score	All parents	75%
2. Proportionate Universal	+0.9SD parenting skills score (intensive); +0.4SD parenting skills score (non-intensive training)	Parents on means-tested benefits (intensive); other parents (non-intensive)	75%
3. Individual risk	+0.9SD parenting skills score (intensive)	Parents living on means-tested benefits	75%
4. Area-based risk	+0.9SD parenting skills score (intensive)	Parents living in the most disadvantaged fifth of residential areas	75%
5. Combination of risks (1)	+0.9SD parenting skills score (intensive)	Families identified according to Troubled Families Programme (TFP) criteria	75%
6. Combination of risks (2)	+0.9SD parenting skills score (intensive)	Mothers meeting Family Nurse Partnership (FNP) criteria	75%
7. Indicated	+0.9SD parenting skills score (intensive)	Families where the child had MHP at an earlier age	75%

Family Nurse Partnership criteria: first-time mothers under 20 years of age.

Troubled Families Programme criteria: two or more risk factors from three domains (health and well-being, joblessness, or domestic violence).

We canvassed views about targeting mechanisms for interventions and uptake levels through a specially-convened meeting of a family research advisory group organised by a national children's charity and with formal and informal conversations with government and non-government policy experts.

The first two scenarios modelled hypothetical effect sizes that might feasibly be achieved through universal parenting skills training programmes offered to all families with young children:

• Universal intervention: Non-intensive parenting skills training offered to all parents, providing an average 0.4 standard deviation (SD) increase in parenting skills (Scenario 1)
• Proportionate universal intervention: Intensive parenting skills training offered to parents on means-tested benefits, providing a 0.9SD increase in parenting skills; and other parents offered non-intensive training (increasing parenting skills by 0.4SD) (Scenario 2)

The subsequent four scenarios modelled different mechanisms to offer intensive parenting skills training providing an average 0.9SD increase in parenting quality only to targeted (or indicated) groups:

• Individual risk: offered to parents in families living on means-tested benefits (Scenario 3).
• Area-based risk: offered to all parents living in the most disadvantaged fifth of residential areas (Scenario 4).
• Combination of risks: 1. Families identified according to Troubled Families Programme (TFP) criteria with two or more risk factors from three domains (health and well-being, joblessness, or domestic violence) (Scenario 5). 2. Mothers meeting the Family Nurse Partnership (FNP) eligibility criteria at the birth of the cohort child (first-time mothers under 20 years of age) (Scenario 6).
• Indicated: Families where the child had MHP at an earlier age (if the cohort child had an SDQ total difficulties score within the abnormal range [a score of 17 or more] at age 3 years) (Scenario 7).

#### 2.3.1 Uptake

For each scenario, we randomly assigned 75% of children eligible according to the targeting criteria to have received the hypothetical intervention (and so increased their parenting quality score), while the scores of the remaining 25% were unchanged. In a sensitivity analysis, we modelled *low uptake* of parenting skills training, where an increase in parenting quality scores was only applied to a randomly selected 33% of eligible children. We also modelled *differential uptake*, so that, of an overall uptake of 75%, a lower uptake (60%) was assigned to eligible children below the poverty line (equivalised family income below 60% of the national median) when the child was 3 years of age compared to those above (83%).

#### 2.3.2 Eligibility

Scenarios 2 and 3: Receipt of means-tested benefits by the child's family at age 3 years.

Scenario 4: Area-based risk (highest quintile of the Index of Multiple Deprivation, based on main residence at age 3 years).

Scenario 5: Two or more factors similar to multidimensional risk criteria developed for the Troubled Families Programme, operationalised using available MCS data at age 3 years:

• Health or well-being: 1. mental health (a parent reporting severe psychological distress [a score of 13 or more on the Kessler-6 scale], or the cohort child scoring within the abnormal range of the SDQ [a score of 17 or more]); 2. a parent with a drug problem (regularly using recreational drugs) or an alcohol problem (drinking every day or drinking to steady their nerves); 3. a parent or child reporting a long-standing illness at 3 years
• No adult in the household is in work (neither the mother or partner, if present, reported being in employment at age 3 years);
• Domestic violence (a question on whether the father or mother had used force at some time in the relationship, answered by the other partner at age 3 years).

Scenario 6: Family Nurse Partnership (FNP) eligibility status: whether the cohort child was the mother's first child, and if the mother was aged under 20 years when the child was born.

Scenario 7: Indicated according to earlier child MHP (an SDQ score within the abnormal scoring range at 3 years).

### 2.4 Analysis

Modelling was carried out using an approach detailed elsewhere (Pearce et al., 2018). First, the association between SECs and child MHP was estimated fitting logistic regression models, with normal range SDQ scores as the reference group versus those in the borderline/abnormal range. Predicted probabilities (and 95% confidence intervals) obtained from these models were used to estimate prevalence of borderline/abnormal scores, overall and in each income quintile (referred to as ‘unadjusted’). Second, the parenting quality variable was entered into the models, together with baseline and intermediate confounders accounted for using inverse probability of treatment weights (IPTWs). The probabilities from these models show the controlled direct effect (CDE) of SECs on child MHP. This CDE is referred to as the ‘observed’ result as it accounts for observed parenting quality in the MCS. Third, the seven intervention scenarios already described were simulated individually by re-estimating the predicted probabilities of child MHP from the previous CDE model (the second stage) after modifying the observed parenting quality variable. To simulate *effectiveness*, the parenting quality score was multiplied by a factor in units of standard deviation to reflect the improvement in parenting skills that might be expected following either standard or intensive parent skills training. In modelling effectiveness, we generated a normal distribution around the chosen effect sizes, reflecting likely variability in the amount of improvement in parenting quality at the level of individual families. Therefore, while the average effect sizes were set to 0.4SD or 0.9SD, the actual increase in any individual's parenting quality score was an amount randomly drawn from the distributions generated around these effect sizes. The increase in parenting quality scores was only assigned to families who were *eligible* for an intervention. For example, in Scenario 2 those in receipt of means-tested benefits were eligible for intensive parenting skills training (increasing observed parenting quality scores by an average of 0.9SD) with the remainder eligible for non-intensive parenting skills training (increasing observed parenting quality scores by an average of 0.4SD). The size of any increase was bounded by the ceiling score of the Pianta CPRS (a score of 75) achieved by 3% of the sample.

In all three stages, summary measures of relative and absolute inequalities were estimated by repeating regression models with income quintile as a continuous term (fitting a linear socio-economic gradient). Relative inequalities were calculated as the ratio of the fitted probabilities of borderline/abnormal scores between the highest and lowest income quintiles (risk ratio [RR]; 95% confidence interval [CI]), and absolute inequalities as the difference between the fitted probabilities of the highest and lowest income quintiles (risk difference [RD] and 95% CI).

IPTWs were trimmed at the 1st and 99th centiles to remove the excessive influence of extreme values on the results and multiplied by an MCS weight (Plewis, 2007), to account for survey design and attrition up to the age five sweep.

Analyses were performed in Stata SE 13.1 (Stata Corporation, Texas, USA).

### 2.5 Working sample

Of the original 18,296 singleton children in the cohort, 3815 children were excluded as they did not participate in the MCS sweeps when the exposure and outcome variables were measured (9 months and 5 years), as were an additional 82 children with missing values on the exposure variable (household income). This resulted in an analytic sample of 14,399 children. To fill in missing information on confounders, mediator, outcome and targeting or indicated variables, multiple imputation by chained equations (Van Buuren, 2012) models were fitted under a missing at random assumption (Sterne et al., 2009) to create twenty datasets, whose results were combined using Rubin's rules. Analyses were weighted to account for attrition to the age five survey.

## 3 Results

### 3.1 Sample characteristics

The characteristics of those with complete data on all variables of interest (Column A); the main analytic (imputed) sample, imputing missing information on confounders, mediator, outcome and targeting or indicated variables (Column B); and the original MCS sample, showing all the data available for a particular variable (Column C), are shown in Table 2. The analytic and original MCS samples were similar in terms of the distributions (or means) of variables included in the analyses; baseline and intermediate confounders, exposure (household income), mediator (parenting quality), outcome (child MHP) and the range of markers defining targeted or indicated interventions. The complete case sample was less likely to include children from disadvantaged SECs, and fewer children with borderline or abnormal SDQ scores. Subsequent results are reported for the main analytic sample only, although there were equivalent findings in the complete case sample (Appendix B: Table B).

**Table 2:** Characteristics of the MCS: comparison across analytic and original samples.

	Weighted∗ % (observed *n*) unless otherwise stated			
	A. Complete case (*n* = 10221)	B. Analytic (imputed) sample (with *M* = 20) (*n* = 14399)		C. Original MCS sample (*n* = 18296)
Household income quintile (measured at age 9 months)				
Highest (1)	23.2 (2151)		19.8	20.1 (2909)
2	22.7 (2242)		20.1	20.0 (3172)
3	21.2 (2140)		20.0	19.9 (3450)
4	18.2 (2001)		20.0	20.0 (4103)
Lowest (5)	14.8 (1687)		20.1	20.1 (4580)
*Not present at relevant sweep*	*NA*		*NA*	*NA*
*Item missing*	*NA*		*NA*	*82*
Sex				
Male	50.6 (5172)		51.0	51.4 (9417)
Female	49.4 (5049)		49.0	49.6 (8879)
*Not present at relevant sweep*	*NA*		*NA*	*NA*
*Item missing*	*NA*		*NA*	*0*
Baseline confounding (reported at age 9 months)				
Ethnicity				
White	93.8 (9464)		88.6	88.5 (15342)
Mixed	0.8 (72)		1.0	1.0 (188)
Indian	1.2 (165)		1.9	1.9 (476)
Pakistani & Bangladeshi	1.6 (230)		4.1	4.2 (1261)
Black or Black British	1.9 (201)		2.8	2.8 (669)
Other	0.7 (89)		1.5	1.6 (346)
*Not present at relevant sweep*	*NA*		*NA*	*NA*
*Item missing*	*NA*		*NA*	*14*
Maternal age at first live birth, years				
<20y	15.8 (1662)		18.9	18.5 (3706)
20-29y	55.2 (5719)		55.6	55.4 (9914)
30-39y	28.6 (2788)		25.1	25.7 (3921)
>39y	0.4 (52)		0.0	0.4 (81)
*Not present at relevant sweep*	*NA*		*NA*	*NA*
*Item missing*	*NA*		*NA*	*674*
Intermediate confounding (reported at age 3 years)				
Family structure				
Both natural parents	83.2 (8505)		80.4	80.7 (11760)
Reconstituted family	2.2 (212)		2.3	2.3 (319)
Lone parent	14.7 (1504)		17.3	17.0 (2471)
*Not present at relevant sweep*	*NA*		*NA*	*3600*
*Item missing*	*NA*		*NA*	*146*
Children in household				
One child	25.2 (2651)		24.8	24.7 (3629)
Two-three children	66.9 (6701)		65.7	65.9 (9403)
Four or more children	7.9 (869)		9.5	9.4 (1547)
*Not present at relevant sweep*	*NA*		*NA*	*3600*
*Item missing*	*NA*		*NA*	*117*
Parent MHP				
No	96.4 (9838)		86.4	95.7 (12505)
Yes	3.6 (383)		4.8	4.3 (594)
*Not present at relevant sweep*	*NA*		*NA*	*3600*
*Item missing*	*NA*		*NA*	*1597*
Household income poverty				
Not poverty	76.2 (7579)		69.6	70.8 (9855)
Poverty	23.8 (2642)		30.4	29.2 (4690)
*Not present at relevant sweep*	*NA*		*NA*	*3600*
*Item missing*	*NA*		*NA*	*151*
Parent drug problems				
No	98.3 (10038)		98.3	98.3 (14447)
Yes	1.8 (183)		1.7	1.7 (249)
*Not present at relevant sweep*	*NA*		*NA*	*3600*
*Item missing*	*NA*		*NA*	*0*
Parent alcohol problems				
*No*	89.6 (9291)		90.4	90.5 (13484)
*Yes*	10.4 (930)		9.6	9.5 (1212)
*Not present at relevant sweep*	*NA*		*NA*	*3600*
*Item missing*	*NA*		*NA*	*0*
Parenting quality (Pianta CPRS, measured at age 3y)				
Pianta CPRS score (mean, SE)	64.5 (0.09)	64.0 (0.09)		64.4 (0.08)
*Not present at relevant sweep*	*NA*	*NA*		*3600*
*Item missing*	*NA*	*NA*		*2685*
Child MHP at age 5y (SDQ)				
Normal SDQ score	91.5 (9307)	88.6		89.0 (12263)
Borderline/abnormal SDQ score	8.5 (914)	11.4		11.0 (1615)
*Not present at relevant sweep*	*NA*	*NA*		*3818*
*Item missing*	*NA*	*NA*		*600*
Variables for targeted/indicated interventions				
Receipt of means-tested benefits (3y)				
No	85.4 (8679)	81.0		81.3 (11721)
Yes	14.6 (1542)	19.0		18.8 (2852)
*Not present at relevant sweep*	*NA*	*NA*		*3600*
*Item missing*	*NA*	*NA*		*123*
Area deprivation (3y)				
Not lowest quintile	85.4 (8674)	78.2		77.8 (10531)
Lowest quintile	14.6 (1547)	21.8		22.1 (4164)
*Not present at relevant sweep*	*NA*	*NA*		*3600*
*Item missing*	*NA*	*NA*		*1*
Troubled Families Programme criteria met (3y)				
No	88.4 (9022)	85.4		86.3 (11031)
Yes	11.6 (1199)	14.6		13.7 (1784)
*Not present at relevant sweep*	*NA*	*NA*		*3600*
*Item missing*	*NA*	*NA*		*1881*
Family Nurse Partnership criteria met (9m)				
No	92.7 (9435)	91.5		91.6 (16529)
Yes	7.4 (786)	8.5		8.4 (1767)
*Not present at relevant sweep*	*NA*	*NA*		*NA*
*Item missing*	*NA*	*NA*		*0*
Child MHP at age 3y (SDQ score within the abnormal range)				
No	92.0 (9376)	85.4		89.7 (12186)
Yes	8.0 (845)	11.1		10.3 (1462)
*Not present at relevant sweep*	*NA*	*NA*		*3600*
*Item missing*	*NA*	*NA*		*1048*

*n*: number of children; *M*: number of imputed subsamples; SE: standard error; NA: Not applicable; SDQ: Strength and Difficulties Questionnaire; CPRS: Child-Parent Relationship Scale; MHP: mental health problems.

Column A: Constrained to complete data on all variables of interest; B: Multiply imputed dataset, imputing missing information on confounders, mediator, outcome and targeting or indicated variables; C: All data available for that variable (unconstrained). Analyses reported in this paper were carried out using the main analytic, imputed dataset (Column B).

∗ To account for sample design and attrition to relevant sweep.

### 3.2 Descriptives

At age 5 years observed prevalence of child MHP, defined as an SDQ total difficulties score within the borderline/abnormal range, was 11%. Child MHP was strongly socially patterned according to household income at 9 months, with both relative and absolute inequalities observed (Table 3: A). Parenting quality (measured using the Pianta CPRS scale) at age 3 years was associated with child MHP at 5 years. On average, Pianta CPRS scores were lower for children with subsequent mental health problems (mean score: 57.6 (95%CI: 57.1–58.2)) compared to other children (64.9 (64.7–65.0)). As with child MHP, Pianta CPRS scores were socially patterned: the mean score for children from the lowest household income quintile was 61.9 (61.5–62.3) compared to 65.3 (65.0–65.6) in the highest income quintile (Appendix C: Table C1).

**Table 3:** Prevalences and relative and absolute income inequalities in child MHP, observed and after modelling parenting skills intervention scenarios.

Prevalence of child MHP according to quintiles of household income									Overall prevalence of child MHP	Inequalities in child MHP (comparing highest and lowest income quintiles)	
1 (highest)	2		3		4		5 (lowest)			Risk difference	Risk ratio
A: UNADJUSTED#											
4.1%	6.9%		8.8%		15.6%		21.3%		11.4%	17.4% (15.7, 19.2)	5.2 (4.3, 6.1)
B: OBSERVED (CONTROLLED DIRECT EFFECT#∗)											
3.5%	7.7%		9.2%		14.5%		19.6%		11.3%	15.8% (13.4, 18.2)	4.8 (3.6, 5.9)
C: UNIVERSAL INTERVENTION SCENARIOS#∗											
Universal increase in Pianta CPRS score (parenting quality) of 0.4SD (Scenario 1)											
2.9%		6.3%		7.5%		12.0%		16.7%	9.4%	13.6% (11.5, 15.7)	5.0 (3.8, 6.2)
Proportionate universal increase in Pianta CPRS score (parenting quality) of 0.9SD if in receipt of means-tested benefits/0.4SD other (Scenario 2)											
2.8%		6.3%		7.4%		11.6%		15.1%	8.9%	12.0% (10.0, 14.0)	4.6 (3.4, 5.7)
D: TARGETED/INDICATED INTERVENTION SCENARIOS#∗											
INDIVIDUAL RISK: Receipt of means-tested benefits (19%): Increase in Pianta CPRS score (parenting quality) of 0.9SD (Scenario 3)											
3.5%		7.6%		9.0%		13.4%		16.3%	10.3%	12.4% (10.3, 14.6)	4.0 (3.0, 4.9)
AREA-BASED RISK: Residing in deprived area (22%): Increase in Pianta CPRS score (parenting quality) of 0.9SD (Scenario 4)											
3.4%		7.5%		8.7%		13.1%		17.1%	10.3%	13.4% (11.2, 15.6)	4.3 (3.3, 5.3)
COMBINATION OF RISKS: Meets Troubled Families Programme criteria (15%): Increase in Pianta CPRS score (parenting quality) of 0.9SD (Scenario 5)											
3.5%		7.5%		8.9%		13.6%		17.3%	10.5%	13.5% (11.3, 15.8)	4.3 (3.3, 5.3)
COMBINATION OF RISKS: Meets Family Nurse Partnership criteria (9%): Increase in Pianta CPRS score (parenting quality) of 0.9SD (Scenario 6)											
3.5%		7.5%		8.9%		14.1%		18.9%	10.9%	15.1% (12.8, 17.4)	4.7 (3.6, 5.8)
INDICATED: SDQ score within abnormal range at 3y (11%): Increase in Pianta CPRS score (parenting quality) of 0.9SD (Scenario 7)											
3.4%		7.3%		8.6%		13.4%		17.9%	10.5%	14.2% (11.9, 16.5)	4.5 (3.4, 5.6)

# Weighted to account for sample design and attrition.

∗ adjusting for: mother's ethnicity, age at first live birth, measured at 9 months; number of children in the household, family structure, parental alcohol problems, parental drug use, parental mental health problems and household income poverty, measured at 3 years.

All potential baseline and intermediate confounders were associated with household income and child MHP, and all confounders except number of children in the household were associated with Pianta CPRS score (Appendix C: Table C2).

Relative and absolute income inequalities in child MHP attenuated after adjustment for potential baseline and intermediate confounding and observed parenting quality (the controlled direct effect, Table 3: B).

### 3.3 Intervention scenarios

Table 3 also shows the expected prevalences and relative and absolute inequalities (comparing highest and lowest income quintiles) in child MHP resulting from the modelled intervention scenarios, based on an assumed 75% uptake of a parenting skills intervention and average effect sizes of 0.4SD (non-intensive) or 0.9SD (intensive), depending on the scenario.

#### 3.3.1 Universal intervention scenarios

Population prevalence and absolute inequalities in child MHP reduced when simulating universal non-intensive (0.4SD) improvements in parenting skills (Table 3: C). However, relative inequalities increased as a consequence of a non-intensive parenting skills intervention for all families (Scenario 1). The provision of more intensive support for poorer families (effect size 0.9SD) was associated with an even greater reduction in prevalence and decreases in both absolute and relative inequalities in child MHP (Scenario 2) (although the latter was small).

#### 3.3.2 Targeted/indicated intervention scenarios

**Fig. 2:**
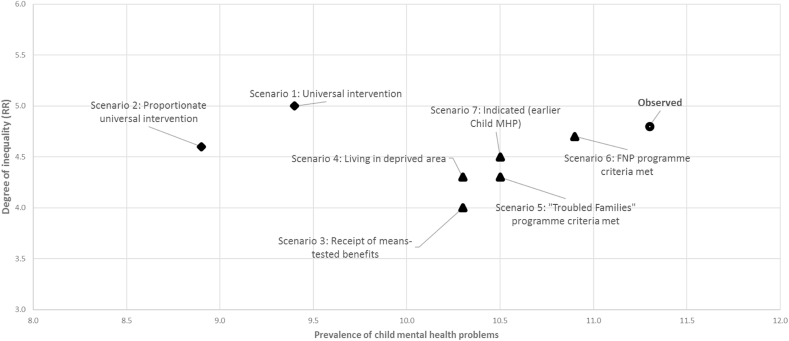
Prevalences and relative income inequalities in child mental health problems according to intervention scenarios.

Compared to universal intervention scenarios, the hypothetical provision of targeted intensive parenting skills training had a smaller impact on overall prevalence, reflecting the relatively small proportions of the population who were eligible for these interventions (Table 3: D). Targeting of families in receipt of benefits (Scenario 3) reduced relative and absolute inequalities in child MHP to a greater extent than a universal non-intensive intervention (Scenario 1), and resulted in comparable absolute, and smaller relative inequalities in child MHP compared to a proportionate universal intervention (Scenario 2). Most other targeted and indicated scenarios reduced inequalities in child MHP (except Family Nurse Partnership criteria, Scenario 6), although the impact was smaller using targeting based on families’ receipt of benefits. Fig. 2 provides a visual comparison for the observed data and each of the Scenarios simulated, plotting overall prevalence versus relative inequalities in child MHP.

#### 3.3.3 Additional sensitivity analyses

We tested a lower effect size of 0.6SD for intensive intervention scenarios, rather than the 0.9SD adopted in the main analyses (Appendix D: Table D1). We also manipulated intervention uptake in all scenarios: an overall uptake of only one third of the sample (Appendix E: Table E1); differential uptake, in which the overall uptake of 75% used in the main analyses masked a lower uptake among less advantaged families than others (Appendix E: Table E2). Although the impact on child MHP prevalences and inequalities of each of the interventions was smaller in these sensitivity analyses than in the main analyses, patterns of results were similar to those reported.

## 4 Discussion

Inequalities in child MHP were apparent at age 5 years in a representative sample of UK children born at the turn of the millennium. In a series of models simulating the effects of hypothetical parenting skills interventions for families with children of 3 years of age or younger, absolute and relative inequalities in child MHP were reduced, but not removed. Scenarios targeting the provision of intensive parenting skills interventions, particularly those that set out to support disadvantaged families, led to the greatest reduction in child MHP inequalities. In contrast, reductions in overall prevalence were more likely to be achieved through universal interventions available to the whole population, even when the simulated effect sizes of interventions were less intensive. A proportionate universal approach (combining intensive parenting skills training for families in receipt of means-tested benefits with less intensive training for others), led to a reduction in both population prevalence and inequality in child MHP, although effect sizes were modest.

### 4.1 Existing literature

We have been able to extend the evidence-base on the association between parenting quality and child MHP. The findings are consistent with those of a number of other studies showing social inequalities in child MHP (Reiss, 2013), including evidence for parenting quality as a mediator between adversity and child MHP (Conger et al., 2010; Linver et al., 2002; Mazza et al., 2016). We showed that the association between socioeconomic circumstances and child MHP was partially explained by parenting quality, as reported in a previous study based on the Millennium Cohort Study (Schoon et al., 2010).

Parenting skills training is now widely recommended in the UK (Davies, 2013; National Institute of Health and Care Excellence, 2012), in order to improve child mental health. A systematic review of qualitative studies on parents’ views about parenting programmes found that they valued the acquisition of knowledge, skills and understanding, together with feelings of acceptance and support from other parents in the parenting group, and that this enabled parents to regain control and feel more able to cope. This led to a reduction in feelings of guilt and social isolation, increased empathy with their children and confidence in dealing with their behaviour (Kane et al., 2007).

Evaluation of parenting skills programmes has been based principally to date on a set of heterogeneous, small trials, which do not address questions of “real world” implementation. Evidence from large-scale evaluations of services supporting families and children, is mixed, and show the results of policy decisions already taken. Our simulation findings demonstrated that parenting skills interventions have the potential to influence child MHP prevalence and inequalities when scaled to a population level under different, realistic scenarios. While this provides information for policy decisions not yet taken to complement those based on empirical evaluations, future implementation strategies would need to take into account other real world issues, such as, for example, the potential need for booster training sessions in order to sustain improvements (Lundahl et al., 2006).

### 4.2 Strengths and limitations

Modelling the potential effects on prevalence and inequalities in child MHP of simulated parenting skills interventions in a representative sample of UK children allowed us to address a number of “What if?” policy questions, which it would not be possible to investigate using trial data alone, including estimating population consequences of each intervention scenario, and how these might differ according to particular targeting strategies or levels of uptake.

A major strength of these analyses is the use of the UK Millennium Cohort Study, a large, nationally-representative contemporary cohort, which provided the opportunity to model a plausible temporal sequence between exposure and outcome, with parenting quality as a mediator measured at the intermediate data collection sweep. In addition, the MCS has rich data on socio-demographic characteristics of children and families, which we were able to use to account for baseline and intermediate confounding, and targeting and indicated variables at the appropriate data collection sweeps. As the exposure variable, we used household income but results were similar when we used highest maternal educational qualification. We had as the outcome the Strengths and Difficulties Questionnaire, a widely-used, validated measure of child MHP (Goodman, 1997). Results from sensitivity analyses for Internalising and Externalising scales, and using the higher abnormal behaviour cut-off, were similar to those reported for the overall Total Difficulties score.

Nevertheless, there were some limitations to our study. With the exception of residential area classification (IMD), all variables used in these analyses were completed by the mother, carrying the potential for report bias. Assessment of parenting quality in the MCS was limited to a single measure, the Pianta child-parent relationships scale. We carried out separate analyses for the two subscales, Conflicts and Closeness, which were similar to the results shown. We did not account for reverse causation, whereby child MHP influences parenting quality. However, we used early child MHP (an SDQ score within the abnormal behaviour range, measured at 3 years) as a potential indicated variable for intensive parenting skills training, and results showed levels of prevalence and inequalities in child MHP that were similar to those from other targeting strategies. Missing values and attrition are always a concern in longitudinal research. Our analyses comprised the initial three data collection sweeps, from 9 months to 5 years of age, with consequently less attrition than would occur over a longer period of time (71% of the singletons in the original sample participated in these three sweeps). We used response weights and multiple imputation by chained equations to account for attrition and item missingness. When complete case analyses were conducted, results were similar to those shown for multiply imputed datasets, suggesting that the findings reported are robust.

The analysis strategy adopted, using marginal structural modelling, only allowed for the inclusion of a single, continuous mediator variable. While we accounted for a wide range of factors, nevertheless the potential for residual confounding remains. In developing hypothetical intervention scenarios, we identified only limited available intervention evidence on parenting quality and child MHP in the pre-school years, particularly from a population perspective, and with a focus on child MHP inequalities. In modelling population interventions, effect sizes were hypothetical extrapolations, drawn primarily from Cochrane reviews and meta-analyses (Barlow et al., 2014; Furlong et al., 2012; Lindsay et al., 2010; Nowak & Heinrichs, 2008), acknowledging that the evidence-base is heterogeneous, and from small trials rather than national programmes. Therefore, as a sensitivity analysis, we modelled a lower effect size for intensive interventions. Uptake of the interventions was modelled at a reasonably high level, at 75%. However, we also modelled a low (33%) uptake, as well as differential uptake for lower income families. Patterns of results from sensitivity analyses for both lower effect size and uptake were similar to those reported.

## 5 Conclusion

These analyses are illustrative of an approach for simulating the national roll out of a hypothetical parenting skills programme that can take into account effectiveness, different approaches to targeting an intervention, and differences in uptake of services, each of which can be modified. Thus, data about real children who are representative of all children in the UK are used to answer “What if” questions about different policy options. From a methodological perspective, the modelling approach adopted (Chittleborough et al., 2014; Pearce et al., 2018) can be easily modified to simulate intervention scenarios, and different exposures, mediators and outcomes at population level, in other settings.

While this study cannot address the practicalities of real-life implementation, our results suggest that a non-intensive, universal parenting skills intervention has the potential to improve overall mental health of children in the UK population. In addition, the modelling exercise showed consistently across scenarios that a targeted approach to the provision of intensive parenting skills interventions might contribute to a reduction in child MHP inequalities, particularly when supporting disadvantaged families identified according to existing administrative criteria, such as receipt of means-tested benefits. However, in every scenario modelled, child MHP inequalities remained, reflecting the fact that the causes of mental health problems are numerous, and that inequalities emerge through a multitude of pathways. This suggests the need for a more comprehensive, upstream approach to tackle the many drivers of inequalities in child MHP, including addressing poverty directly.

## Ethical statement

Ethical approval was received from a Research Ethics Committee at each study survey. The present secondary data analyses did not require additional ethics approval.

## Funding

This work is based on independent research commissioned and funded by the 10.13039/501100000272National Institute for Health Research Policy Research Programme. The views expressed in this publication are those of the author(s) and not necessarily those of the NHS, the National Institute for Health Research, the Department of Health and Social Care or its arm's length bodies, and other Government Departments. Anna Pearce is supported by funds from the 10.13039/100010269Wellcome Trust [205412/Z/16/Z], the 10.13039/501100000265Medical Research Council [MC_UU_12017/13] and the 10.13039/100014589Scottish Government Chief Scientist Office [SPHSU13]. Research at UCL Great Ormond Street Institute of Child Health and Great Ormond Street Hospital for Children receives a proportion of the funding from the 10.13039/501100000272Department of Health’s NIHR Biomedical Research Centres funding scheme.

## CRediT authorship contribution statement

**Steven Hope:** Conceptualization, Methodology, Formal analysis, Data curation, Writing – original draft, Writing – review & editing, Visualization. **Anna Pearce:** Conceptualization, Methodology, Writing – original draft, Writing – review & editing. **Mario Cortina-Borja:** Methodology, Writing – original draft, Writing – review & editing. **Catherine Chittleborough:** Methodology, Writing – original draft, Writing – review & editing. **Jane Barlow:** Writing – original draft, Writing – review & editing. **Catherine Law:** Conceptualization, Methodology, Writing – original draft, Writing – review & editing.
