# Supplementary material for: Modelling the potential for parenting skills interventions to reduce inequalities and population prevalence of children's mental health problems: Evidence from the Millennium Cohort Study
Source: SSM Popul Health. 2021 May 13;14:100817. doi: 10.1016/j.ssmph.2021.100817 (PMC8209401; doi:10.1016/j.ssmph.2021.100817)
Supplement: Multimedia component 1 [file mmc1.docx]

**Modelling the potential for parenting skills interventions to reduce inequalities and population prevalence of children’s mental health problems: Evidence from the Millennium Cohort Study**

**SUPPLEMENTARY APPENDICES**

**APPENDIX A: Alternative measure of socio-economic circumstances: maternal education**

**TABLE A1: Prevalences and relative and absolute inequalities in child MHP, observed and after modelling parenting skills intervention scenarios (75% uptake)**

| **Prevalence of child MHP:**  **according to maternal education~** | | **Overall prevalence**  **of child MHP** | **Inequalities in child MHP (comparing high and low education level)** | |
| --- | --- | --- | --- | --- |
| High  Education | Low  education |  | Risk difference | Risk ratio |
| **OBSERVED (CONTROLLED DIRECT EFFECT ^#^*****)** | | | | |
| **7.8%** | **19.5%** | **11.0%** | **11.8% (10.0, 13.5)** | **2.5 (2.2, 2.8)** |
| **UNIVERSAL INTERVENTION SCENARIOS^#^*** | | | | |
| Universal increase in Pianta CPRS score (parenting quality) of 0.4SD **(Scenario 1)** | | | | |
| 6.4% | 16.6% | 9.2% | 10.2% (8.9, 11.8) | 2.6 (2.3, 2.9) |
| Proportionate universal increase in Pianta CPRS score (parenting quality) of 0.9SD if in receipt of means-tested benefits / 0.4SD other **(Scenario 2)** | | | | |
| 6.2% | 15.6% | 8.8% | 9.4% (7.9, 10.9) | 2.5 (2.2, 2.8) |
| **TARGETED/INDICATED INTERVENTION SCENARIOS ^#^*** | | | | |
| INDIVIDUAL RISK: Receipt of means-tested benefits: Increase in Pianta CPRS score (parenting quality) of 0.9SD **(Scenario 3)** | | | | |
| 7.4% | 17.5% | 10.2% | 10.0% (8.5, 11.7) | 2.4 (2.1, 2.6) |
| AREA-BASED RISK: Residing in deprived area: Increase in Pianta CPRS score (parenting quality) of 0.9SD **(Scenario 4)** | | | | |
| 7.3% | 17.3% | 10.0% | 10.0% (8.4, 11.7) | 2.4 (2.1, 2.7) |
| COMBINATION OF RISKS: Meets Troubled Families Programme criteria: Increase in Pianta CPRS score (parenting quality) of 0.9SD **(Scenario 5)** | | | | |
| 7.4% | 18.0% | 10.3% | 10.5% (8.9, 12.2) | 2.4 (2.1, 2.7) |
| COMBINATION OF RISKS: Meets Family Nurse Partnership criteria: Increase in Pianta CPRS score (parenting quality) of 0.9SD **(Scenario 6)** | | | | |
| 7.5% | 18.9% | 10.7% | 11.3% (9.6, 13.0) | 2.5 (2.2, 2.8) |
| INDICATED: SDQ score within abnormal range at 3y: Increase in Pianta CPRS score (parenting quality) of 0.9SD **(Scenario 7)** | | | | |
| 7.3% | 17.8% | 10.2% | 10.5% (8.8, 12.2) | 2.4 (2.1, 2.7) |

~Highest academic qualification, at least GCSE grade A-C or higher vs lower or none

^#^Weighted to account for sample design and attrition.

*Adjusting for: mother’s ethnicity, age at first live birth, measured at 9 months; number of children in the household, family structure, parental alcohol problems, parental drug use, parental mental health problems and household income poverty, measured at 3 years

**APPENDIX B: Complete case analysis**

**TABLE B1: Prevalences and relative and absolute inequalities in child MHP, observed and after modelling parenting skills intervention scenarios (complete case analysis [n=10,221], 75% uptake)**

| **Prevalence of child MHP**  **according to quintiles of household income** | | | | | | | | **Overall prevalence**  **of child MHP** | | **Inequalities in child MHP (comparing highest and lowest income quintiles)** | |
| --- | --- | --- | --- | --- | --- | --- | --- | --- | --- | --- | --- |
| 1 (highest) | | 2 | | 3 | | 4 | 5 (lowest) |  |  | Risk difference | Risk ratio |
| **OBSERVED (CONTROLLED DIRECT EFFECT^#^*****)** | | | | | | | | | | | |
| **3.1%** | | **5.9%** | | **7.7%** | | **11.4%** | **14.0%** | **8.1%** | | **11.0% (8.6, 13.4)** | **4.1 (2.9, 5.2)** |
| **UNIVERSAL INTERVENTION SCENARIOS^#^*** | | | | | | | | | | | |
| Universal increase in Pianta CPRS score (parenting quality) of 0.4SD **(Scenario 1)** | | | | | | | | | | | |
| 2.6% 2.5% | | 4.8% | | 6.3% | | 9.5% | 11.8% | 6.7% | | 9.3% (7.3, 11.4) | 4.2 (3.0, 5.4) |
| Proportionate universal increase in Pianta CPRS score (parenting quality) of 0.9SD if in receipt of means-tested benefits / 0.4SD other **(Scenario 2)** | | | | | | | | | | | |
| 2.5% | | 4.7% | | 6.3% | | 9.2% | 10.8% | 6.4% | | 8.3% (6.4, 10.3) | 3.9 (2.8, 5.0) |
| **TARGETED/INDICATED INTERVENTION SCENARIOS^#^*** | | | | | | | | | | | |
| INDIVIDUAL RISK: Receipt of means-tested benefits: Increase in Pianta CPRS score (parenting quality) of 0.9SD **(Scenario 3)** | | | | | | | | | | | |
| 3.1% | | 5.8% | | 7.6% | | 10.8% | 11.7% | 7.5% | | 8.6% (6.5, 10.8) | 3.4 (2.4, 4.4) |
| AREA-BASED RISK: Residing in deprived area: Increase in Pianta CPRS score (parenting quality) of 0.9SD **(Scenario 4)** | | | | | | | | | | | |
| 3.1% | | 5.8% | | 7.3% | | 10.4% | 12.5% | 7.5% | | 9.4% (7.2, 11.6) | 3.7 (2.6, 4.7) |
| COMBINATION OF RISKS: Meets Troubled Families Programme criteria: Increase in Pianta CPRS score (parenting quality) of 0.9SD **(Scenario 5)** | | | | | | | | | | | |
| 3.1% | | 5.8% | | 7.5% | | 10.8% | 12.4% | 7.6% | | 9.3% (7.1, 11.6) | 3.7 (2.6, 4.7) |
| COMBINATION OF RISKS: Meets Family Nurse Partnership criteria: Increase in Pianta CPRS score (parenting quality) of 0.9SD **(Scenario 6)** | | | | | | | | | | | |
| 3.1% | 5.8% | | 7.6% | | 11.1% | | 13.5% | 7.9% | | 10.4% (8.1, 12.8) | 3.9 (2.8, 5.0) |
| INDICATED: SDQ score within abnormal range at 3y: Increase in Pianta CPRS score (parenting quality) of 0.9SD **(Scenario 7)** | | | | | | | | | | | |
| 3.0% | 5.7% | | 7.3% | | 10.7% | | 13.0% | 7.6% | 10.0% (7.7, 12.3) | | 3.9 (2.8, 5.0) |

^#^Weighted to account for sample design and attrition.

*Adjusting for: mother’s ethnicity, age at first live birth, measured at 9 months; number of children in the household, family structure, parental alcohol problems, parental drug use, parental mental health problems and household income poverty, measured at 3 years

**APPENDIX C: Additional associations between variables of interest in the analytic sample**

**TABLE C1: Mean Pianta CPRS** **score at 3 years according to income quintile at 9 months (weighted)**

| **Weekly Household Income** | **Weighted Mean Pianta CPRS (95% CI)** |
| --- | --- |
| Highest quintile (1) | 65.3 (65.0-65.6) |
| 2 | 65.0 (64.7-65.3) |
| 3 | 64.5 (64.2-64.8) |
| 4 | 63.5 (63.2-63.9) |
| Lowest quintile (5) | 61.9 (61.5-62.3) |
| *p*-value | <0.001 |

**TABLE C2: Associations between baseline and intermediate confounders with household income, child MHP and Pianta CPRS score (% or mean, weighted)**

|  | **Weekly Household Income at 9m** | **Child MHP at 5 years** | **Pianta CPRS score at 3 years** |
| --- | --- | --- | --- |
|  | *Weighted mean, £s* | *Weighted % SDQ borderline/abnormal score* | *Weighted mean* |
| **Baseline confounders (9m)** |  |  |  |
| *Maternal age at first live birth*, years (categorised) |  |  |  |
| < 20y | 165.13 | 18.7 | 62.5 |
| 20-29y | 303.77 | 11.2 | 64.2 |
| 30-39y | 474.41 | 6.2 | 64.9 |
| >39y | 492.62 | 12.8 | 64.9 |
| *p*-value | <0.001 | <0.001 | <0.001 |
| *Ethnicity* |  |  |  |
| White | 332.46 | 10.5 | 64.1 |
| Mixed | 251.77 | 16.7 | 63.6 |
| Indian | 325.41 | 13.8 | 65.0 |
| Pakistani/Bangladeshi | 176.67 | 22.7 | 61.8 |
| Black/Black British | 231.45 | 18.6 | 64.8 |
| Other (including Chinese) | 267.61 | 8.9 | 63.9 |
| *p*-value | <0.001 | <0.001 | <0.001 |
| **Intermediate confounders (3y)** |  |  |  |
| *Family structure* |  |  |  |
| Both natural parents | 356.29 | 9.0 | 64.5 |
| Reconstituted family | 196.18 | 18.1 | 61.7 |
| Lone parent | 174.50 | 21.5 | 62.4 |
| *p*-value | <0.001 | <0.001 | <0.001 |
| *Children in household* |  |  |  |
| One child | 331.89 | 12.3 | 63.8 |
| Two-three children | 332.74 | 10.4 | 64.1 |
| Four or more children | 213.43 | 15.2 | 64.2 |
| *p*-value | <0.001 | <0.001 | 0.20 |
| *Parent MHP* |  |  |  |
| No | 326.19 | 10.3 | 64.3 |
| Yes | 213.73 | 34.5 | 58.0 |
| *p*-value | <0.001 | <0.001 | <0.001 |

| *Household income* |  |  |  |
| --- | --- | --- | --- |
| Not poverty | 391.52 | 7.5 | 64.8 |
| Poverty | 159.98 | 20.1 | 62.3 |
| *p*-value | <0.001 | <0.001 | <0.001 |
| *Parent alcohol problems* |  |  |  |
| No | 316.18 | 11.5 | 64.0 |
| Yes | 368.88 | 9.9 | 64.0 |
| *p*-value | <0.001 | 0.13 | 0.72 |
| *Parent drug problems* |  |  |  |
| No | 322.64 | 11.2 | 64.1 |
| Yes | 239.97 | 18.1 | 62.5 |
| *p*-value | <0.001 | <0.01 | <0.01 |

**APPENDIX D: Modelling alternative effect size for intensive intervention scenarios**

**TABLE D1: Prevalences and relative and absolute inequalities in child MHP, observed and after modelling parenting skills intervention scenarios (effect size for intensive interventions: 0.6SD)**

| **Prevalence of child MHP**  **according to quintiles of household income** | | | | | | | | | **Overall prevalence**  **Of child MHP** | | | **Inequalities in child MHP (comparing highest and lowest income quintiles)** | |
| --- | --- | --- | --- | --- | --- | --- | --- | --- | --- | --- | --- | --- | --- |
| 1 (highest) | 2 | | 3 | | 4 | | 5 (lowest) | |  |  |  | Risk difference | Risk ratio |
| **OBSERVED (CONTROLLED DIRECT EFFECT ^#^*****)** | | | | | | | | | | | | | |
| **3.5%** | **7.7%** | | **9.2%** | | **14.5%** | | **19.6%** | | **11.3%** | | | **15.8% (13.4, 18.2)** | **4.8 (3.6, 5.9)** |
| **UNIVERSAL INTERVENTION SCENARIOS^#^*** | | | | | | | | | | | | | |
| Universal increase in Pianta CPRS score (parenting quality) of 0.4SD **(Scenario 1)** | | | | | | | | | | | | | |
| 2.9% | | 6.3% | | 7.5% | | 12.0% | | 16.7% | | 9.4% | 13.6% (11.5, 15.7) | | 5.0 (3.8, 6.2) |
| Proportionate universal increase in Pianta CPRS score (parenting quality) of 0.6SD if in receipt of means-tested benefits / 0.4SD other **(Scenario 2)** | | | | | | | | | | | | | |
| 2.8% | | 6.3% | | 7.5% | | 11.9% | | 15.9% | | 9.2% | 12.9% (10.9, 15.0) | | 4.8 (3.7, 6.0) |
| **TARGETED/INDICATED INTERVENTION SCENARIOS^#^*** | | | | | | | | | | | | | |
| INDIVIDUAL RISK: Receipt of means-tested benefits (19%): Increase in Pianta CPRS score (parenting quality) of 0.6SD **(Scenario 3)** | | | | | | | | | | | | | |
| 3.5% | | 7.6% | | 9.0% | | 13.7% | | 17.1% | | 10.5% | 13.3% (11.1, 15.6) | | 4.2 (3.2, 5.2) |
| AREA-BASED RISK: Residing in deprived area (22%): Increase in Pianta CPRS score (parenting quality) of 0.6SD **(Scenario 4)** | | | | | | | | | | | | | |
| 3.5% | | 7.5% | | 8.8% | | 13.4% | | 17.8% | | 10.5% | 14.0% (11.7, 16.3) | | 4.4 (3.4, 5.5) |
| COMBINATION OF RISKS: Meets Troubled Families Programme criteria (15%): Increase in Pianta CPRS score (parenting quality) of 0.6SD **(Scenario 5)** | | | | | | | | | | | | | |
| 3.5% | | 7.5% | | 8.9% | | 13.8% | | 17.9% | | 10.7% | 14.1% (11.8, 16.4) | | 4.4 (3.4, 5.5) |
| COMBINATION OF RISKS: Meets Family Nurse Partnership criteria (9%): Increase in Pianta CPRS score (parenting quality) of 0.6SD **(Scenario 6)** | | | | | | | | | | | | | |
| 3.5% | | 7.5% | | 9.0% | | 14.2% | | 19.1% | | 11.0% | 15.3% (13.0, 17.7) | | 4.7 (3.6, 5.8) |
| INDICATED: SDQ score within abnormal range at 3y (11%): Increase in Pianta CPRS score (parenting quality) of 0.6SD **(Scenario 7)** | | | | | | | | | | | | | |
| 3.4% | | 7.4% | | 8.7% | | 13.6% | | 18.4% | | 10.7% | 14.7% (12.4, 17.0) | | 4.6 (3.5, 5.7) |

^#^ Weighted to account for sample design and attrition

* adjusting for: mother’s ethnicity, age at first live birth, measured at 9 months; number of children in the household, family structure, parental alcohol problems, parental drug use, parental mental health problems and household income poverty, measured at 3 years

**APPENDIX E: Modelling alternative uptake scenarios**

**TABLE E1: Prevalences and relative and absolute inequalities in child MHP, observed and after modelling parenting skills intervention scenarios (33% uptake)**

| **Prevalence of child MHP**  **according to quintiles of household income** | | | | | | **Overall prevalence**  **of child MHP** | **Inequalities in child MHP (comparing highest and lowest income quintiles)** | |
| --- | --- | --- | --- | --- | --- | --- | --- | --- |
| 1 (highest) | 2 | | 3 | 4 | 5 (lowest) |  | Risk difference | Risk ratio |
| **OBSERVED (CONTROLLED DIRECT EFFECT ^#^*****)** | | | | | | | | |
| **3.5%** | **7.7%** | | **9.2%** | **14.5%** | **19.6%** | **11.3%** | **15.8% (13.4, 18.2)** | **4.8 (3.6, 5.9)** |
| **UNIVERSAL INTERVENTION SCENARIOS^#^*** | | | | | | | | |
| Universal increase in Pianta CPRS score (parenting quality) of 0.4SD **(Scenario 1)** | | | | | | | | |
| 3.2% | 7.1% | | 8.4% | 13.4% | 18.3% | 10.5% | 14.9% (12.6, 17.1) | 4.9 (3.7, 6.1) |
| Proportionate universal increase in Pianta CPRS score (parenting quality) of 0.9SD if in receipt of means-tested benefits / 0.4SD other **(Scenario 2)** | | | | | | | | |
| 3.2% | 7.1% | | 8.4% | 13.2% | 17.6% | 10.2% | 14.1% (11.9, 16.3) | 4.7 (3.6, 5.8) |
| **TARGETED/INDICATED INTERVENTION SCENARIOS^#^*** | | | | | | | | |
| INDIVIDUAL RISK: Receipt of means-tested benefits: Increase in Pianta CPRS score (parenting quality) of 0.9SD **(Scenario 3)** | | | | | | | | |
| 3.5% | 7.6% | | 9.1% | 14.1% | 18.1% | 10.8% | 14.2% (12.0, 16.5) | 4.4 (3.4, 5.4) |
| AREA-BASED RISK: Residing in deprived area: Increase in Pianta CPRS score (parenting quality) of 0.9SD **(Scenario 4)** | | | | | | | | |
| 3.5% | 7.6% | | 9.0% | 13.9% | 18.6% | 10.9% | 14.8% (12.5, 17.2) | 4.6 (3.5, 5.7) |
| COMBINATION OF RISKS: Meets Troubled Families Programme criteria: Increase in Pianta CPRS score (parenting quality) of 0.9SD **(Scenario 5)** | | | | | | | | |
| 3.5% | 7.6% | | 9.0% | 14.1% | 18.5% | 10.9% | 14.7% (12.4, 17.0) | 4.5 (3.4,5.6) |
| COMBINATION OF RISKS: Meets Family Nurse Partnership criteria: Increase in Pianta CPRS score (parenting quality) of 0.9SD **(Scenario 6)** | | | | | | | | |
| 3.5% | 7.6% | 9.0% | | 14.3% | 19.3% | 11.1% | 15.5% (13.1, 17.9) | 4.8 (3.6, 5.9) |
| INDICATED: SDQ score within abnormal range at 3y: Increase in Pianta CPRS score (parenting quality) of 0.9SD **(Scenario 7)** | | | | | | | | |
| 3.5% | 7.6% | 8.9% | | 14.0% | 18.9% | 10.9% | 15.1% (12.8, 17.5) | 4.7 (3.6, 5.8) |

^#^Weighted to account for sample design and attrition.

*Adjusting for: mother’s ethnicity, age at first live birth, measured at 9 months; number of children in the household, family structure, parental alcohol problems, parental drug use, parental mental health problems and household income poverty, measured at 3 years

**TABLE E2: Prevalences and relative and absolute inequalities in child MHP, observed and after modelling parenting skills intervention scenarios (differential uptake: overall 75% uptake, comprising 60% low income group and 83% other income groups)**

| **Prevalence of child MH problems by income**  **according to quintiles of household income** | | | | | **Overall prevalence**  **of child MHP** | **Inequalities in child MH problems (comparing highest and lowest income quintiles)** | |
| --- | --- | --- | --- | --- | --- | --- | --- |
| 1 (highest) | 2 | 3 | 4 | 5 (lowest) |  | Risk difference | Risk ratio |
| **OBSERVED (CONTROLLED DIRECT EFFECT ^#^*****)** | | | | | | | |
| **3.5%** | **7.7%** | **9.2%** | **14.5%** | **19.6%** | **11.3%** | **15.8% (13.4, 18.2)** | **4.8 (3.6, 5.9)** |
| **UNIVERSAL INTERVENTION SCENARIOS^#^*** | | | | | | | |
| Universal increase in Pianta CPRS score (parenting quality) of 0.4SD **(Scenario 1)** | | | | | | | |
| 2.8% | 6.1% | 7.5% | 12.1% | 17.0% | 9.5% | 14.0% (11.9, 16.2) | 5.2 (4.0, 6.5) |
| Proportionate universal increase in Pianta CPRS score (parenting quality) of 0.9SD if in receipt of means-tested benefits / 0.4SD other **(Scenario 2)** | | | | | | | |
| 2.8% | 6.1% | 7.4% | 11.7% | 15.6% | 9.1% | 12.8% (10.8, 14.8) | 4.9 (3.7, 6.0) |
| **TARGETED/INDICATED INTERVENTION SCENARIOS^#^*** | | | | | | | |
| INDIVIDUAL RISK: Receipt of means-tested benefits: Increase in Pianta CPRS score (parenting quality) of 0.9SD **(Scenario 3)** | | | | | | | |
| 3.5% | 7.6% | 9.0% | 13.6% | 16.8% | 10.4% | 13.0% (10.8, 15.2) | 4.1 (3.1, 5.1) |
| AREA-BASED RISK: Residing in deprived area: Increase in Pianta CPRS score (parenting quality) of 0.9SD **(Scenario 4)** | | | | | | | |
| 3.4% | 7.5% | 8.6% | 13.0% | 17.5% | 10.3% | 13.7% (11.5, 15.9) | 4.3 (3.3, 5.4) |
| COMBINATION OF RISKS: Meets Troubled Families Programme criteria: Increase in Pianta CPRS score (parenting quality) of 0.9SD **(Scenario 5)** | | | | | | | |
| 3.4% | 7.5% | 8.9% | 13.7% | 17.7% | 10.6% | 13.9% (11.7, 16.2) | 4.4 (3.3, 5.4) |
| COMBINATION OF RISKS: Meets Family Nurse Partnership criteria: Increase in Pianta CPRS score (parenting quality) of 0.9SD **(Scenario 6)** | | | | | | | |
| 3.5% | 7.5% | 9.0% | 14.1% | 19.1% | 11.0% | 15.3% (12.9, 17.6) | 4.7 (3.6, 5.8) |
| INDICATED: SDQ score within abnormal range at 3y: Increase in Pianta CPRS score (parenting quality) of 0.9SD **(Scenario 7)** | | | | | | | |
| 3.4% | 7.3% | 8.5% | 13.4% | 18.1% | 10.5% | 14.4% (12.1, 16.7) | 4.6 (3.5, 5.6) |

^#^Weighted to account for sample design and attrition.

*Adjusting for: mother’s ethnicity, age at first live birth, measured at 9 months; number of children in the household, family structure, parental alcohol problems, parental drug use, parental mental health problems and household income poverty, measured at 3 years
